# Mycorrhizal Inoculation Differentially Affects Grapevine's Performance in Copper Contaminated and Non-contaminated Soils

**DOI:** 10.3389/fpls.2018.01906

**Published:** 2019-01-25

**Authors:** Amaia Nogales, Erika S. Santos, Maria Manuela Abreu, Diego Arán, Gonçalo Victorino, Helena Sofia Pereira, Carlos M. Lopes, Wanda Viegas

**Affiliations:** ^1^Linking Landscape, Environment, Agriculture and Food (LEAF), Instituto Superior de Agronomia (ISA), Universidade de Lisboa, Lisbon, Portugal; ^2^Instituto de Investigaciones Tecnológicas, University of Santiago de Compostela, Santiago de Compostela, Spain; ^3^Improyen Consulting, Santa Comba, Spain

**Keywords:** arbuscular mycorrhizal fungi, symbiosis, *Vitis vinifera* L. *cv*. touriga nacional, copper, soil contamination, manganese toxicity

## Abstract

Plant inoculation with arbuscular mycorrhizal fungi (AMF) is increasingly employed to enhance productivity and sustainability in agricultural ecosystems. In the present study, the potential benefits of AMF inoculation on young grapevines replanted in pots containing vineyard soil with high Cu concentration were evaluated. For this purpose, one-year-old *cv*. Touriga Nacional grapevines grafted onto 1103P rootstocks were further inoculated with *Rhizoglomus irregulare* or *Funneliformis mosseae*, or left non-inoculated, and maintained in a sterilized substrate under greenhouse conditions for three months. After this time, half of the plants were transplanted to containers filled with an Arenosol from a vineyard which had been artificially contaminated or not with 300 mg kg^−1^ of Cu. At the end of the growing season, soil nutrient concentration, soil dehydrogenase activity and mycorrhizal colonization rate were analyzed. Grapevine performance was assessed by measuring several vegetative growth and physiological parameters as well as nutrient concentrations in leaves and roots. In the non-contaminated soil, *R. irregulare*- and *F. mosseae*-inoculated plants had significantly greater root biomass than the non-inoculated ones. However, the opposite effect was observed in the Cu-contaminated soil, where non-inoculated plants performed better regarding shoot and root development. Concerning nutrient levels, an increase in Cu, Mg and Mn concentrations was observed in the roots of plants growing in the contaminated soil, although only Mn was translocated to leaves. This led to a large increase in leaf Mn concentrations, which was significantly higher in non-inoculated and *F. mosseae*- inoculated plants than in the *R. irregulare*- inoculated ones. Copper contamination induced a general decrease in leaf N, P and Fe concentrations as well as chlorosis symptoms. The largest decrease in N and P was observed in *F. mosseae*- inoculated plants, with 73 and 31.2%, respectively. However, these plants were the ones with the least decrease in Fe concentration (10% vs. almost 30% in the other two inoculation treatments). In conclusion, this study indicates that soil Cu levels can modify the outcome of AMF inoculations in young grapevines, disclosing new AMF-plant associations potentially relevant in vineyards with a tradition of Cu-based fungicide application.

## Introduction

Copper is an essential micronutrient for most organisms. In plants, it has important functions in key physiological processes, and is usually taken up by root systems in the form of divalent cations or Cu chelates from the available fraction of soils. Relevant plant processes which require Cu include photosynthetic electron transport, mitochondrial respiration, oxidative stress responses, cell wall metabolism and hormone signaling (Marschner, 1995; Raven et al., 1999; Kabata-Pendias, 2010; Miotto et al., 2014). Copper is also a structural element in regulatory proteins, acting as a cofactor in many metalloproteins (Yruela, 2009). However, it can be highly toxic for plants and soil (micro)organisms at high concentrations (Leita et al., 1995; Adrees et al., 2015).

Repeated application of Cu-based fungicides in vineyards since the end of the 19th century has led to a significant increase in the total concentration of Cu in many viticultural soils (Magalhães et al., 1985; Brun et al., 1998; Besnard et al., 2001; Arias et al., 2004; Casali et al., 2008; Mackie et al., 2012). Since Cu is scarcely mobile in soils, it commonly remains concentrated in surface soil horizons, decreasing its concentration along the soil profile (Magalhães et al., 1985; Kabata-Pendias, 2010). However, several agricultural practices such as tillage may contribute to the presence of Cu in deeper layers. In practice, the presence of Cu in the surface layers of soil is not expected to induce phytotoxicity in mature grapevines, whose root systems extend deeply in the soil (Anne and Dupuis, 1953). In contrast, young vines have much shallower root systems, and may therefore be affected by high surface soil Cu concentrations (Conradie, 2004), which can lead to phytotoxicity events in vine nurseries or in recently replanted vineyards (Giovannini, 1997; Conradie, 2004; Melo et al., 2008).

Although Cu availability in the soil depends on factors such as soil texture, pH, redox potential and organic matter content (Fageria et al., 2002), it has been observed that Cu first accumulates in the roots in some plant species (Brun et al., 2001), reducing root growth in terms of dry biomass, length, as well as branch number (Lequeux et al., 2010). Furthermore, Cu can induce root cuticle damage (Sheldon and Menzies, 2005) and root cracks (Michaud et al., 2008), which may decrease water and nutrient absorption capacity and thereby alter tissue nutrient levels and ultimately affect plant growth (Kopittke et al., 2009). Plant exposure to high Cu concentrations and its potential translocation and accumulation in shoots may lead to disrupted plant development, changes in nutrient contents, decreased chlorophyll levels in leaves, and photosynthesis inhibition (Yruela, 2005, 2009; Kabata-Pendias, 2010). Other negative effects include delayed flowering and fruiting (Brun et al., 2003; Jin et al., 2015), reduction in plant survival (Brun et al., 2003), and Cu translocation to grapes and wines (Vystavna et al., 2014; Sun et al., 2018). This can be harmful to the health of consumers, especially if other elements such as Fe, Zn, Ni, Pb, and Sc are present (Araya et al., 2003; Turnlund et al., 2004; Naughton and Petróczi, 2008). However, since plant species influence rhizosphere conditions, and consequently the availability of elements as well as their uptake (Bonfante and Genre, 2010), increased Cu concentrations in the soil do not necessarily lead to an increase of this element in plant tissues/organs.

Besides its effects on plants, high Cu concentrations are also toxic to soil organisms. Copper accumulation in soil can severely decrease total microbial biomass as well as the functional diversity of the soil microbial community, reducing decomposition rates of organic matter and impairing specific pathways of nutrient cycling (Kandeler et al., 1996; Fernández-Calviño et al., 2010; Mackie et al., 2012, 2013). However, some soil microorganisms, including arbuscular mycorrhizal fungi (AMF), have developed adaptive mechanisms for their survival and growth in environments with high Cu concentrations (reviewed in Ferrol et al., 2009).

Arbuscular mycorrhizal fungi are obligate symbionts that colonize more than 80% of land plants (Schüβler et al., 2001). The symbiotic association is based on the exchange of nutrients between fungal partners and plants, where AMF provide the host plant with water and nutrients that would otherwise be inaccessible, and plants provide AMF with photosynthetic products (Smith and Read, 2008). In addition to an improved nutritional supply, mycorrhizal symbiosis confers numerous advantages to the plant, including increased growth and productivity and enhanced tolerance to various biotic and abiotic stresses (e.g., Pozo and Azcón-Aguilar, 2007; Smith and Read, 2008; Porcel et al., 2012; Maya and Matsubara, 2013; Augé et al., 2015). Mycorrhizal fungi can also have an important role in alleviating metal toxicity in plants (Carvalho et al., 2006; Hildebrandt et al., 2007; Ferrol et al., 2009; Ambrosini et al., 2015; Jin et al., 2015). In particular, some AMF isolates have been reported to decrease Cu bioavailability in contaminated soils by excreting glomalin related proteins, a type of glycoproteins, or by storing Cu in cellular compartments with reduced metabolism, including spores and vesicles (reviewed in Ferrol et al., 2009). Moreover, the ability of AMF species to mitigate the stress caused by high soil Cu contents in plants have been attributed to a promotion on the absorption of water and nutrients, particularly P, through the roots (Soares and Siqueira, 2008; Andrade et al., 2010). The enhancement of plant antioxidant enzyme activities by AMF and the consequent decrease of oxidative damage to lipids also contributes to better plant survival in Cu-contaminated soils (Merlos et al., 2016).

In grapevines, the effect of plant inoculation with AMF in soils with high Cu concentration varies greatly depending on the species, as seen in Ambrosini et al. (2015). In this work, the authors tested the effects of six different AMF species on young 1103P grapevine rootstock plants and observed that although all AMF species induced an accumulation of Cu in roots, *Rhizophagus clarus* and *Rhizophagus irregulare* inoculated plants had increased dry root biomass. Also, P levels were generally improved in shoots and roots, except in *Destisculata heterogama* and *Acaulospora colombiana* inoculated plants.

In view of the scarcity of data on the effect of AMF on young grapevine tolerance to high Cu concentrations, and of the importance of this issue for grapevine nurseries and for the replanting of new grapevines in old vineyards, in the present study the main objective was to study the effects of AMF inoculation in several growth, physiological and nutritional parameters in grapevines grown in an Arenosol artificially contaminated with Cu.

## Materials and Methods

### Biological Material and Soil Characteristics

Thirty dormant vines of Touriga Nacional *cv*. grafted onto 1103 Paulsen rootstock were obtained from a Portuguese plant nursery. Plant roots were washed with tap water and cut to 2 cm before being planted in 600 mL forest pot containers filled with autoclave-sterilized (40 min at 120°C in two consecutive days) substrate mixture of *Sphagnum* peat: perlite (2:1, *V*:*V*). The pH value of the substrate was corrected to 6.5 with Ca(CO)_3._ Grapevines were inoculated with AMF at planting time by placing the inoculum in the planting hole and in contact with the roots, as follows: 10 plants were inoculated with 10 g of *Rhizoglomus irregulare* (Błaszk., Wubet, Renker & Buscot) Sieverd., G.A. Silva & Oehl BEG-72 inoculum (≈100 infective propagules per gram of carrier material) provided by the Agrifood Institute of Research and Technology, IRTA (Barcelona, Spain) and 10 plants were inoculated with 20 g of *Funneliformis mosseae* (T.H. Nicolson & Gerd.) C. Walker & A. Schüßler inoculum (≈55 infective propagules per gram) obtained from Symbiom company (Czech Republic). The remaining 10 plants were left as non-inoculated control plants, where 20 g of the carrier material (without AMF) was applied. Grapevines were kept under greenhouse conditions for three months and daily watered. One gram of slow release fertilizer based on NPK (16:8:16 + Micronutrients, Bayfolan Multi, Bayer) was applied in each container during this period.

After the three months growth period, shoot length and the number of leaves were recorded in all plants and root mycorrhizal colonization percentage was estimated in three composite samples from each treatment by the gridline intersect method (Giovannetti and Mosse, 1980) after staining them with 0.05% trypan blue in lactic acid (Phillips and Hayman, 1970; Koske and Gemma, 1989). Plants were then transplanted to a soil that had been previously collected from the “Central Experimental Pole for the Conservation of Autochthonous Grapevine Genetic Variability-PORVID” in Pegões, Portugal. This soil had not been cultivated for the last six years, and prior to that it was planted with orange and plum trees. Before the experiment was set, the soil from Pegões was thoroughly homogenized, and soil samples were collected for chemical and biological analysis.

### Experimental Setup

A total of 120 kg of air-dried soil (fraction <5 mm) from Pegões was split into two fractions. The first one (60 kg) was contaminated with 300 mg kg^−1^ of Cu (in the form of CuSO_4_·5H_2_O) and left for four weeks in plastic bags at room temperature in the dark. The other 60 kg were stored at identical conditions during the same period.

In July 2016, three months after inoculation, half of the non-inoculated grapevine plants, *R. irregulare*-inoculated plants and *F. mosseae*-inoculated plants were transplanted to containers filled with 4 kg of Cu-contaminated soil and the other half of the plants of each inoculation treatment were transplanted to containers filled with 4 kg of non-contaminated soil. Plants were left under open air conditions until the end of the growing season (beginning of October) and fertilized twice with an aqueous solution containing NH_4_NO_3_, KNO_3_ and KH_2_PO_4_ to ensure the contribution of 500 mg of N and K and 50 mg of P.

During the growing season, plant performance was monitored by measuring the normalized difference vegetation index (NDVI), which is an important indicator of chlorophyll content in plants, and the photochemical reflectance index (PRI), a useful indicator of the physiological status of plants (Trotter et al., 2002; Filella et al., 2009). Additionally, in August, plant total leaf area was estimated following the non-destructive method of Lopes and Pinto (2005) adapted to greenhouse-grown young grapevine plants.

At the end of the growing season, three months after transplant, plants were harvested and shoot length and fresh root biomass were measured. Root and leaf samples were collected from each plant for chemical analysis and for the evaluation of mycorrhizal colonization. Furthermore, soil samples were collected from each container for chemical and biological analysis.

### Plant Chemical Analysis

For leaf chemical analysis, 30 leaves were collected, washed with distilled water, dried at 40°C until constant weight was achieved and finely grounded. Although petiole mineral analysis is commonly used in mature grapevines to assess their nutritional state (Fallahi et al., 2005; Romero et al., 2010), the amount of petiole tissue in the young grapevines was not sufficient for nutrient analysis and therefore, the complete leaf tissue, including blades and petioles, was used.

For root analysis, roots were washed with distilled water and separated into two parts. One part was stored for posterior determination of root mycorrhizal colonization rate as described above, and the other part was cut and sonicated in distilled water in an ultrasound bath for 30 min. Then, they were subsequently dried at 40°C until constant weight and finely grounded.

Elements (except N) from leaf and root samples (3 grams per sample) were extracted with ultrapure concentrated HCl 3 M after calcining at 500°C for 6 h. Phosphorus was determined in this solution by visible spectrophotometry using the molybdenum blue method (EPA method 365.1. O'Dell, 1993) while Ca, Cu, Fe, K, Mg, Mn, Na, and Zn were analyzed by flame atomic absorption spectroscopy. Total concentration of N was analyzed by Kjeldahl method (Póvoas and Barral, 1992).

### Biological and Chemical Analysis of Soils

The number of mycorrhizal infective propagules and soil dehydrogenase activity were determined in three fresh soil samples collected from Pegões. The number of mycorrhizal infective propagules was estimated by the most probable number (MPN) technique (Porter, 1979; Powell, 1980) using leeks (*Allium porrum* L.) as trap plants, that grew during six months under greenhouse conditions. Then, leek roots were stained with a Trypan blue dye (Phillips and Hayman, 1970; Koske and Gemma, 1989) and the number of infective propagules in the soil was calculated using the mathematical model of Jarvis et al. (2010). Furthermore, overall microbial activity was assessed by analyzing dehydrogenase activity in another three fresh soil samples (< 2 mm fraction), using the triphenyltetrazolium chloride method as described by Tabatabai (1994), using a solution of 0.1M of TRIS buffer (pH 7.6).

Further analysis, including pH and electric conductivity in H_2_O (1:2.5 *m*:*V*), organic C by wet combustion and cation exchange capacity (CEC) by 1 mol L^−1^ ammonium acetate at pH 7, were carried out on three other dried soil samples (<2 mm fraction) (Póvoas and Barral, 1992). Nitric and ammoniacal N were analyzed following the methodology of Mulvaney (1996), and total concentrations of Ca, P, Fe, K, Mg, Na, Cu, Mn, and Zn were determined by atomic emission spectrometry with induced plasma and instrumental neutron activation analysis after acid digestion with HClO_4_+HNO_3_+HCl+HF (Activation Laboratories Ltd, Canada).

Furthermore, after three months grapevine growth in this soil (contaminated or non-contaminated with 300 mg kg^−1^ of Cu), soil pH and soil extractable concentrations of Fe, Cu, Mn, and Zn were determined by previously extracting them in a aqueous solution of diethylenetriaminepentaacetic acid (DTPA) (0.005 mol L^−1^ DTPA + 0.1 mol L^−1^ triethanolamine + 0.01 mol L^−1^ calcium chloride; Lindsay and Norvell, 1978). The elements were analyzed by flame atomic absorption spectroscopy. In addition, soil dehydrogenase activity was also measured as described above.

### Statistical Analysis

Statistical analyses were performed using the SPSS Statistics vs. 23 (IBM) software. All the data obtained were tested for normality and homogeneity of variance.

Vegetative growth parameters (number of leaves per plant and shoot length) and mycorrhizal colonization rates in grapevines grown for three months in the sterile substrate were analyzed by a one-way ANOVA test. Differences among groups were determined by a Tukey b *post hoc* test (*p* ≤ 0.05).

After grapevine transplant to the Arenosol, soil and plant parameters were analyzed by a two-way ANOVA where Cu contamination and Mycorrhizal inoculation treatment were considered as main factors. Differences among groups were determined by a Tukey b *post hoc* test (*p* ≤ 0.05). In cases where data did not have a normal distribution or homogeneous variance, Kruskall-Wallis test followed by Dunn-Bonferroni multiple pairwise comparison test (*p* ≤ 0.05) was implemented.

## Results

### Mycorrhizal Colonization and Grapevine Growth in Sterilized Substrate

**Figure 1:**
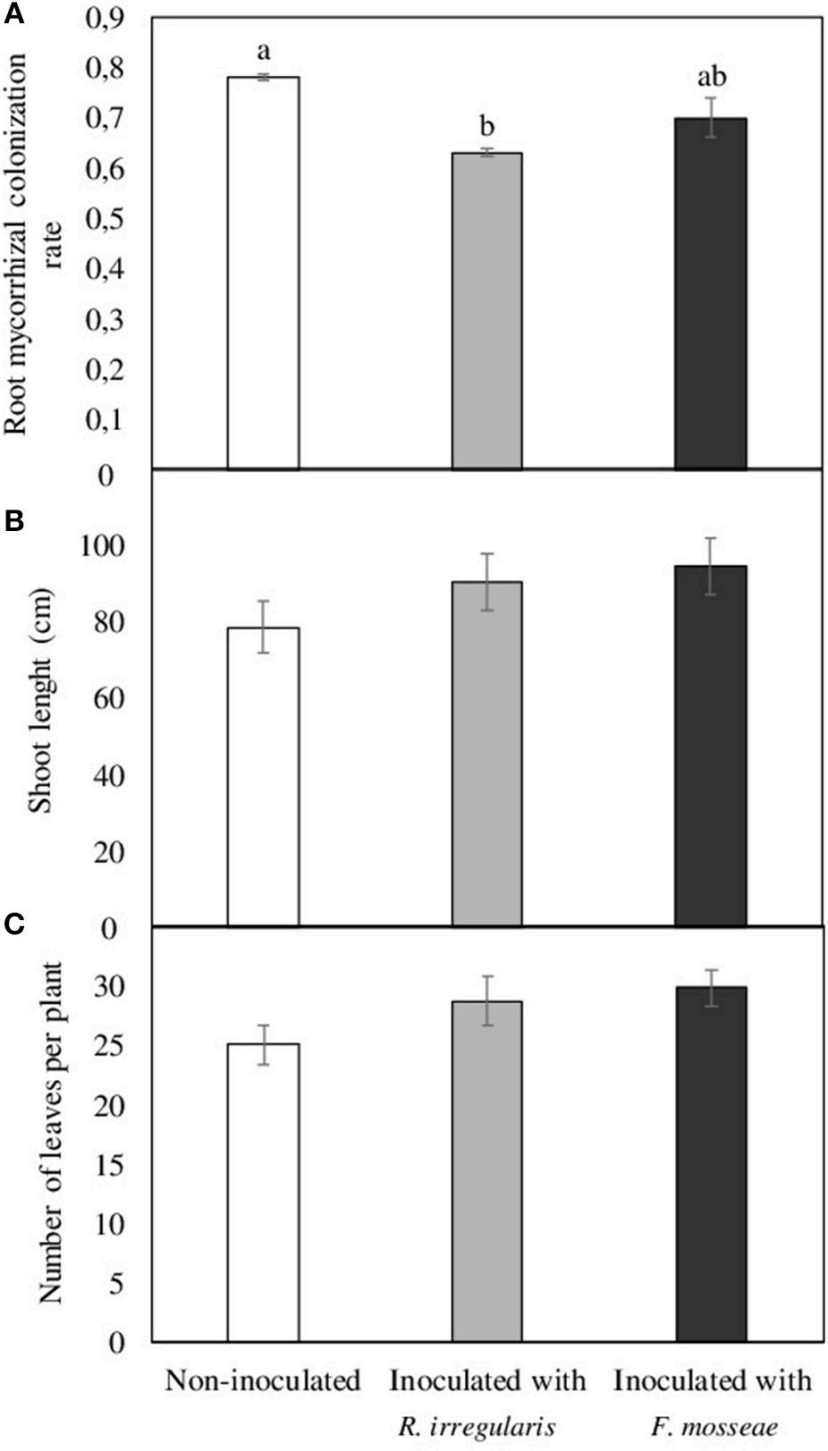
**(A)** Root mycorrhizal colonization rate, **(B)** shoot length, and **(C)** number of leaves per plant in Touriga Nacional grapevine variety plants grafted onto 1103 Paulsen rootstock three months after inoculation and transplant to forest pot containers filled with sterile substrate. Data represent average values of three composite samples per treatment ± standard error in **(A)**, and 10 plants ± standard error in **(B,C)**. Different letters indicate significant differences among treatments according to Tukey b *post-hoc* tests at *p* ≤ 0.05.

As demonstrated in Figure 1A, the three months period after inoculation in previously sterilized substrate resulted in well-established mycorrhizal symbiosis, with root colonization rates of 0.63 and 0.70 in *R. irregulare*- and *F. mosseae*-inoculated plants, respectively. Mycorrhizal colonization was also observed in non-inoculated plants, which was even higher than in the inoculated ones (0.78, Figure 1A). Regarding plant development in the aerial part, no differences were observed in shoot length and leaf number (Figures 1B,C).

### Experimental Soil Characteristics

The soil collected from Pegões and used for the experiments was an Arenosol (I. U. S. S., Working Group WRB—World Reference Base for Soil Resources, 2015) and had neutral pH, very low fertility (as indicated by the contents of organic C and the two forms of mineral N) as well as low electrical conductivity and CEC (Table 1). It contained 6.4 mycorrhizal infective propagules per gram of soil (Table 1).

**Table 1:** Physico-chemical and biological parameters of the soil used in the experiment.

**Texture**	**Sandy**
**PHYSICO-CHEMICAL CHARACTERISTCS**	
pH (H_2_O)	7.0 ± 0.03
Electric conductivity (μS cm^−1^)	73.0 ± 2.70
Cation exchange capacity (cmol(+) kg^−1^)	3.06 ± 0.170
Organic C (g kg^−1^)	5.7 ± 0.213
N-NO_3_ (mg kg^−1^)	1.11 ± 0.336
N-NH_4_ (mg kg^−1^)	1.86 ± 0.142
**TOTAL NUTRIENT CONCENTRATION (mg kg**^**−1**^**)**	
Ca	933.3 ± 27.22
P	263.3 ± 7.20
Fe	2400.0 ± 216.02
K	22066.7 ± 803.23
Mg	400.0 ± 0.0
Na	967.3 ± 27.21
Cu	26.67 ± 1.186
Mn	77.00 ± 5.735
Zn	14.33 ± 1.440
**BIOLOGICAL CHARACTERISTCS**	
Number of mycorrhizal propagules (g^−1^)	6.4
Dehydrogenase activity (mg TPF.g^−1^)	11.9 ± 0.90

*Values represent the average of 3 samples ± standard error*.

Soil characteristics were significantly altered after the experimental period. No significant interaction was detected among the factors Cu contamination and Mycorrhizal inoculation treatment in pH or in any of the analyzed elements in the soil extractable fraction (Fe, Cu, Zn, and Mn- Table 2A). However, as expected, Cu contamination had a significant effect on extractable Cu concentration, which increased more than 41-fold, as well as on Fe and Mn, that increased 1.7 and 1.3-fold in average, respectively (Table 2B). pH in the contaminated soil decreased while Mn concentration increased. Zinc concentration was not affected by Cu contamination (Table 2), and the effect of mycorrhizal inoculation was not significant in any of these element concentrations (Table 2).

**Table 2:** **(A)** *P*-values of the two-way ANOVA test for the effects of the factors “Copper contamination” and “Mycorrhizal inoculation treatment” and their interaction; **(B)** soil pH and micronutrient concentration in extractable fraction of the soil after three months growth of Touriga Nacional grapevine variety plants grafted onto 1103P rootstock inoculated or not with *R. irregulare* or *F. mosseae*.

**(A) Factor**		**Soil pH**	**Fe**	**Cu**	**Zn**	**Mn**
Copper contamination		< 0.0001	< 0.0001	< 0.0001	0.076	0.005
Mycorrhizal inoculation treatment		0.357	0.686	0.658	0.489	0.353
Interaction		0.343	0.573	0.161	0.390	0.503
**(B) Soil treatment**	**Inoculation treatment**	**Soil pH**	**Micronutrient concentration in the extractable fraction (mg kg**^**−1**^**)**			
			**Fe**	**Cu**	**Zn**	**Mn**
Non-contaminated soil	Non-inoculated	7.2 ± 0.06 a	12.12 ± 0.733 b	2.68 ± 0.119 b	1.79 ± 0.240 a	1.96 ± 0.128 a
	Inoculated with *R. irregulare*	7.3 ± 0.09 a	15.71 ± 1.875 ab	3.03 ± 0.201 b	1.89 ± 0.435 a	1.84 ± 0.109 a
	Inoculated with *F. mosseae*	7.3 ± 0.04 a	12.49 ± 1.045 b	2.46 ± 0.191 b	1.63 ± 0.150 a	1.91 ± 0.157 a
Cu-contaminated soil	Non-inoculated	6.8 ± 0.07 b	22.44 ± 1.944 a	128.37 ± 4.910 a	2.68 ± 0.338 a	2.75 ± 0.321 a
	Inoculated with *R. irregulare*	6.9 ± 0.04 b	22.51 ± 2.698 a	125.16 ± 10.760 a	1.89 ± 0.165 a	2.39 ± 0.205 a
	Inoculated with *F. mosseae*	6.7 ± 0.04 b	23.49 ± 2.267 a	131.99 ± 2.560 a	2.19 ± 0.286 a	2.20 ± 0.152 a

*Data represent average values (n = 5) ± standard error. Different letters indicate significant differences according to Tukey b a posteriori test. Significant effect: p-value ≤ 0.05*.

Copper contamination had also a significant effect on soil dehydrogenase activity (Table 3A), which showed a decrease in the soil with high Cu concentration (Table 3B). However, the effect of mycorrhizal inoculation treatments was not significant (Table 3A).

**Table 3:** **(A)** *P*-values of the two-way ANOVA for the effects of the factors “Copper contamination” and “Mycorrhizal inoculation treatment” and their interaction; **(B)** Soil dehydrogenase activity (in mg TPF g^−1^ dry weight) and root mycorrhizal colonization rate in Touriga Nacional plants grafted onto 1103 Paulsen rootstock at the end of the growing season.

**(A) Factor**	**Soil dehydrogenase activity**		**Root mycorrhizal colonization rate**	
Copper contamination	<0.0001		0.744	
Mycorrhizal inoculation treatment	0.287		0.575	
Interaction	0.567		0.919	
**(B) Inoculation treatment**	**Soil dehydrogenase activity**		**Root mycorrhizal colonization rate**	
	**Non-contaminated soil**	**Cu-contaminated soil**	**Non-contaminated soil**	**Cu-contaminated soil**
Non-inoculated	13.80 ± 1.386 a	0.67 ± 0.086 b	0.54 ± 0.069 a	0.49 ± 0.070 a
Inoculated with *R. irregulare*	12.34 ± 0.812 a	0.49 ± 0.069 b	0.55 ± 0.034 a	0.59 ± 0.026 a
Inoculated with *F. mosseae*	13.64 ± 1.689 a	0.50 ± 0.069 b	0.51 ± 0.059 a	0.52 ± 0.053 a

*Data represent average values (n = 5) ± standard error. Different letters indicate significant differences among treatments according to Tukey b a posteriori tests at p-value ≤ 0.05*.

### Effects of Mycorrhizal Inoculation and Cu Contamination on Plant Fitness

Root mycorrhizal colonization at the end of the growing season (in October) ranged from 0.49 to 0.59 and was not affected neither by Cu contamination nor by the mycorrhizal inoculation treatment (Table 3).

**Figure 2:**
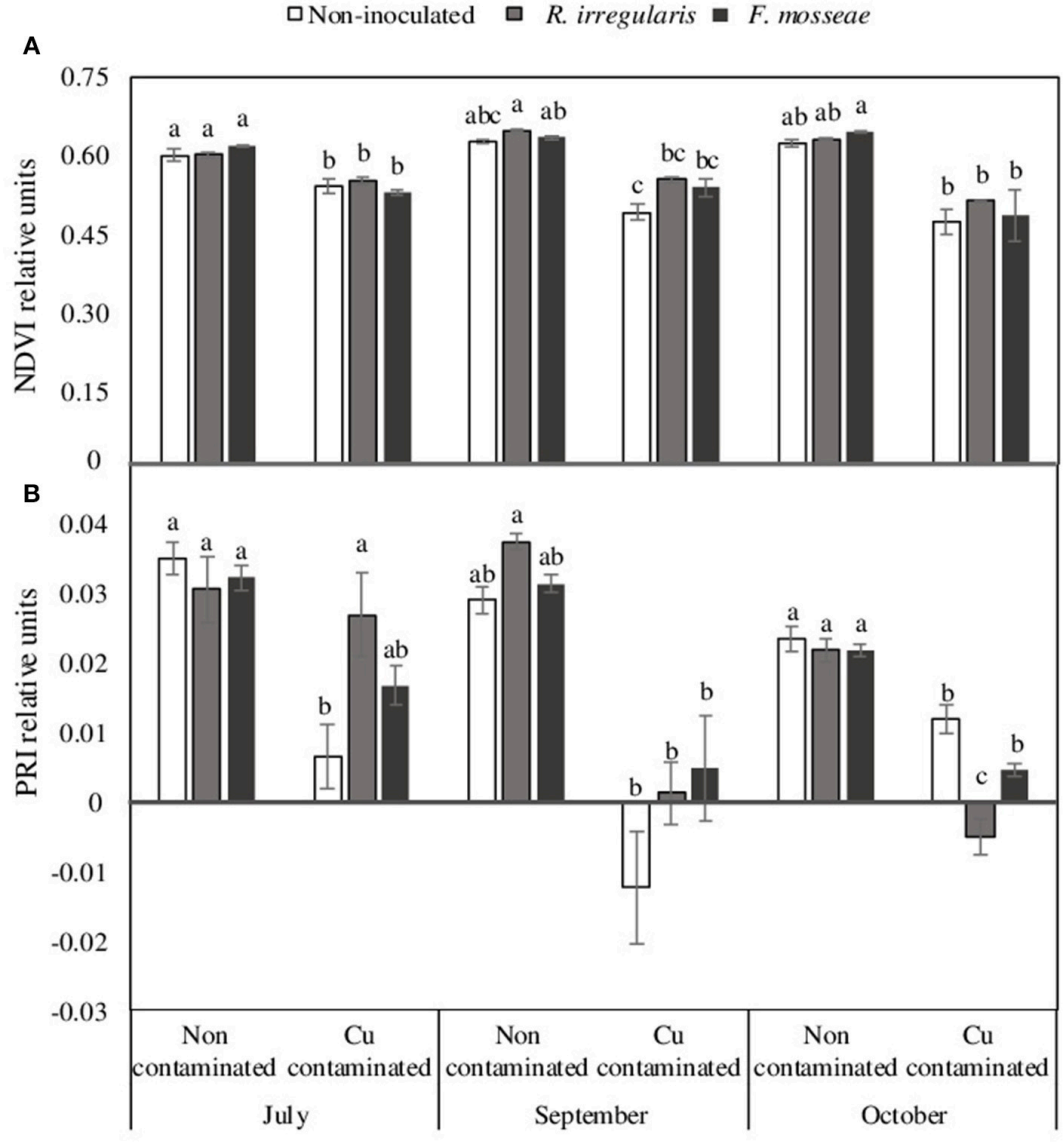
**(A)** Normalized difference vegetation index, NDVI and **(B)** Photochemical reflectance index, PRI, data of Touriga Nacional grapevine variety plants grafted onto 1103 Paulsen rootstock growing in Cu-contaminated or non-contaminated soil at different time points during the growing season. Bars indicate the average of five plants per treatment ± standard error. Different letters indicate significant differences at each month (July, September or October) according to Tukey b or Dunn-Bonferroni tests at *p* ≤ 0.05.

**Figure 3:**
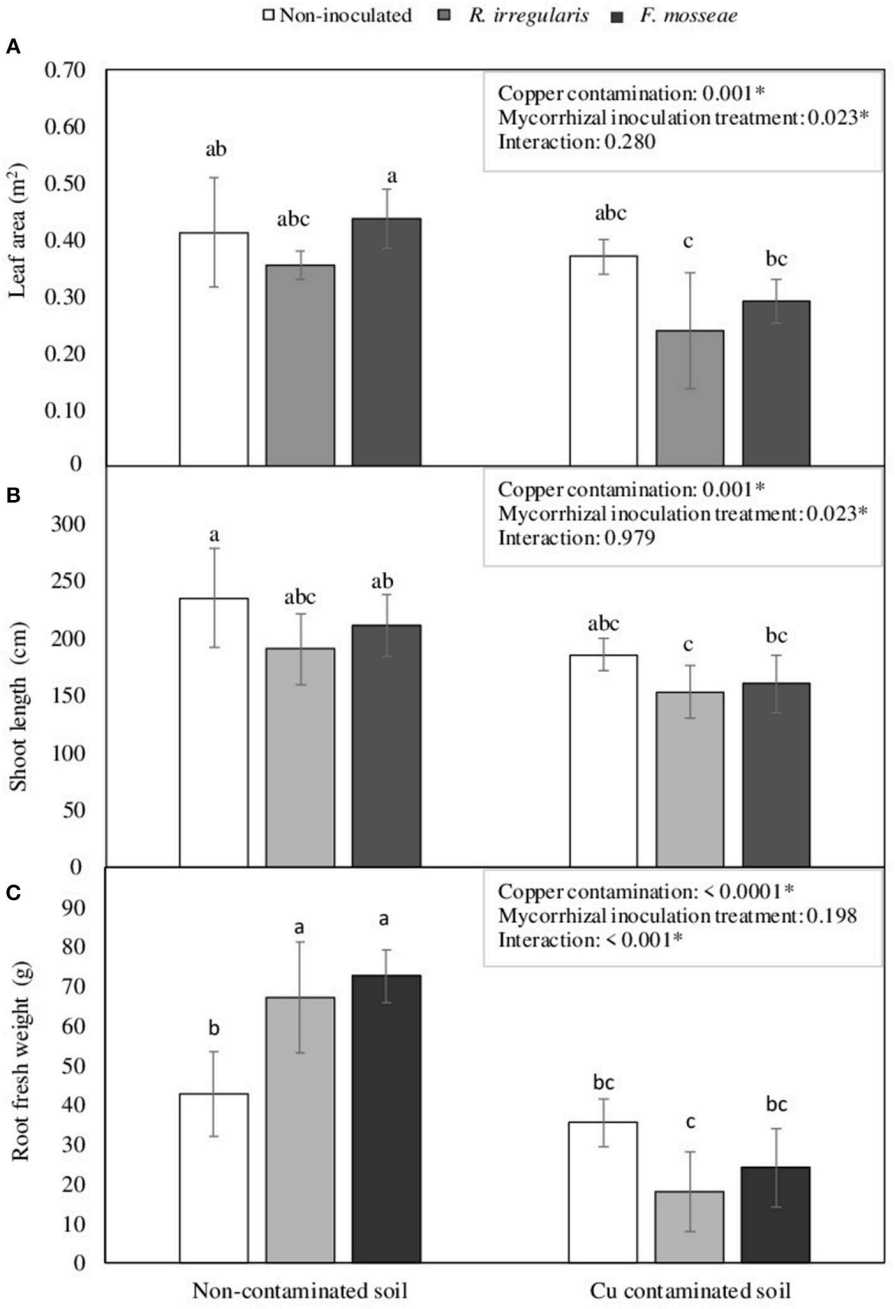
**(A)** Leaf area, **(B)** shoot length, and **(C)** root fresh weight in Touriga Nacional grapevine variety plants grafted onto 1103P rootstock inoculated or not with *R. irregulare* or *F. mosseae*. Each bar represents the average value of five plants ± standard error. Different letters indicate significant differences according to Tukey b *post-hoc* test. The box inside the graphics indicate the significance values of the two-way factorial ANOVA with interaction where Copper contamination and Mycorrhizal inoculation treatment were considered as main factors.

However, although the different mycorrhizal inoculation treatments did not have a significant effect in NDVI, PRI, leaf area and shoot length in plants grown in the non-contaminated soil (Figures 2, 3), these parameters were affected by high Cu concentration in the soil.

In the Cu-contaminated soil, starting in July, just two weeks after grapevine transplant, a significant decrease in NDVI index was already observed in all mycorrhizal inoculation treatments (Figure 2A). By October, this decrease was only statistically significant in *F. mosseae*-inoculated plants (Figure 2A). Concerning PRI, in July only non-inoculated plants had a significant decrease due to Cu contamination (Figure 2B), but in October, all three inoculation treatments showed a significant decrease on this index, which was lowest in *R. irregulare*-inoculated plants (Figure 2B).

By the end of the experimental period, Cu contamination in the soil induced a general decrease in total leaf area and shoot length (Figures 3A,B). Total plant leaf area was reduced in 32.4 and 33.5% in *R. irregulare*- and *F. mosseae*- inoculated plants, respectively, while non-inoculated plants showed a decrease of only 10.3%. The decrease in shoot length was similar for all three inoculation treatments, ranging from 19.6 to 24.2%.

**Figure 4:**
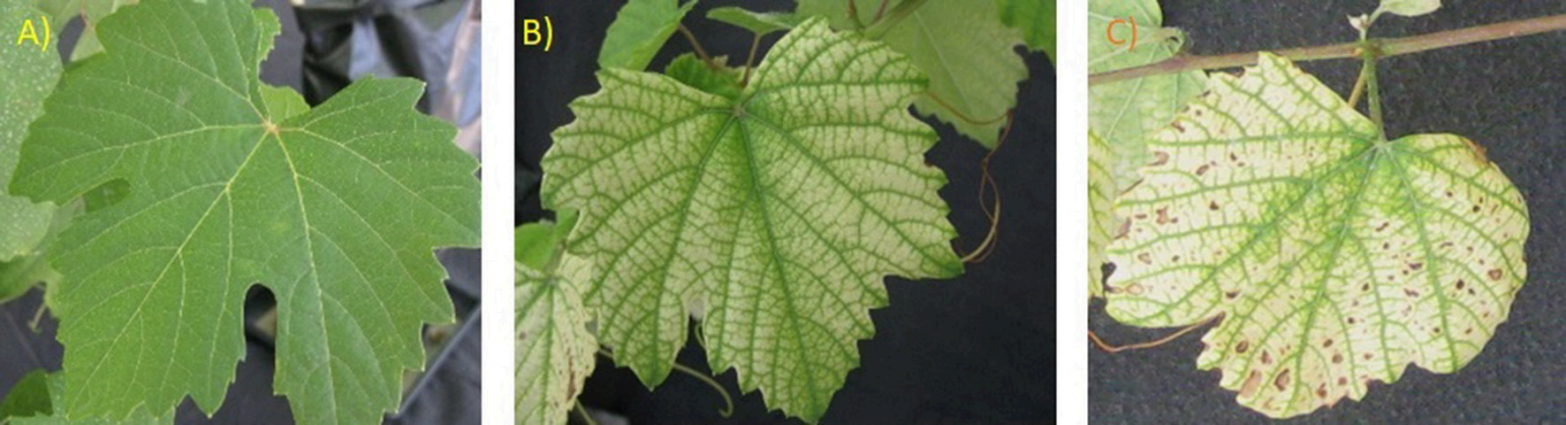
**(A)** Non-symptomatic leaf of a grapevine plant grown in the non-contaminated Arenosol. **(B)** Interveinal chlorosis symptoms in a grapevine plant growing in the Cu-contaminated Arenosol. **(C)** Chlorosis and dark spots in a grapevine plant growing in the Cu-contaminated Arenosol.

Plants grown at high soil Cu concentrations presented leaves with interveinal chlorosis, that in some cases (mostly in the non-inoculated plants) were accompanied by dark spots (Figure 4). Those symptoms started two weeks after transplant in non-inoculated plants, and later, in September, they were also observed in AMF-inoculated plants. Symptomatic leaves were mainly the youngest ones. At the end of the growing season, non-inoculated plants presented 70% of symptomatic leaves, while *R. irregulare*- and *F. mosseae*- inoculated plants presented 51 and 59% of symptomatic leaves, respectively. Nonetheless, due to the high variability in this parameter within the same treatment, statistical differences were not significant (data not shown).

Concerning root biomass, a significant interaction was found between Cu contamination and Mycorrhizal inoculation treatment factors. In the non-contaminated soil, AMF inoculated plants had significantly higher root biomass than non-inoculated ones (Figure 3C). However, in the Cu-contaminated soil, these plants were the ones showing the largest decrease, 73.1 and 66.8%, respectively (Figure 3C), while the non-inoculated ones kept relatively unchanged root biomass.

### Effects of AMF Inoculation and Cu on Plant Nutritional Status

At the end of the growing season, after the three months experimental period, in the non-contaminated soil, nutrient concentration in roots did not differ among mycorrhizal inoculation treatments (Table 4). In leaves, only P concentration showed a significant effect of the mycorrhizal inoculation treatment, being *F. mosseae*-inoculated plants the ones showing the highest P levels, followed by non-inoculated and by *R. irregulare*- inoculated plants (Table 5).

**Table 4:** **(A)** *P*-values of the two-way ANOVA test for the effects of the factors “Copper contamination” and “Mycorrhizal inoculation treatment” and their interaction; **(B)** Macro- and micronutrient contents in roots of Touriga Nacional grapevine variety plants grafted onto 1103P rootstock inoculated or not with *R. irregulare* or *F. mosseae*.

**(A) Factor**		**Macronutrient**						**Micronutrient**			
		**N**	**P**	**K**	**Na**	**Ca**	**Mg**	**Fe**	**Cu^1^**	**Zn^1^**	**Mn**
Copper contamination		0.603	0.652	0.002	0.067	0.064	< 0.0001	0.017	–	–	< 0.0001
Mycorrhizal inocualtion treatment		0.283	0.125	0.003	0.044	0.029	0.215	0.012	–	–	0.963
Interaction		0.496	0.572	0.040	0.575	0.025	0.722	0.002	–	–	0.857
**(B) Soil treatment**	**Inoculation treatment**	**Macronutrient concentraction (g kg**^**−1**^**)**						**Micronutrient concentration (mg kg**^**−1**^**)**			
		**N**	**P**	**K**	**Na**	**Ca**	**Mg**	**Fe**	**Cu**^1^	**Zn**^1^	**Mn**
Non-contaminated	Non-inoculated	8.28 ± 1.179 a	2.21 ± 0.129 a	5.32 ± 0.292 b	1.81 ± 0.348 b	5.41 ± 0.531 a	1.04 ± 0.114 bc	79.36 ± 11.286 b	< DL ^2^	< DL ^2^	27.87 ± 1.667 b
	*R. irregulare*	8.30 ± 0.686 a	2.57 ± 0.072 a	6.28 ± 0.387 b	2.33 ± 0.171 ab	5.91 ± 0.325 a	0.86 ± 0.043 c	76.51 ± 1.963 b	< DL ^2^	< DL ^2^	27.14 ± 1.106 b
	*F. mosseae*	8.72 ± 0.733 a	2.41 ± 0.079 a	5.94 ± 0.263 b	2.47 ± 0.287 ab	6.94 ± 0.258 a	0.92 ± 0.053 c	125.24 ± 11.166 b	< DL ^2^	< DL ^2^	28.84 ± 2.770 b
Cu-contaminated soil	Non-inoculated	7.57 ± 0.858 a	2.38 ± 0.108 a	6.77 ± 0.510 b	2.23 ± 0.163 ab	6.49 ± 0.870 a	1.58 ± 0.134 a	85.49 ± 12.549 b	91.02 ± 11.402 a	26.51 ± 0.906 b	60.96 ± 7.823 a
	*R. irregulare*	8.54 ± 0.230 a	2.50 ± 0.134 a	9.69 ± 0.804 a	2.97 ± 0.299 a	2.56 ± 0.396 b	1.47 ± 0.174 a	268.45 ± 59.287 a	134.78 ± 21.142 a	36.68 ± 3.422 a	69.50 ± 11.519 a
	*F. mosseae*	10.59 ± 1.989 a	2.44 ± 0.118 a	6.14 ± 0.945 b	2.58 ± 0.163 ab	5.68 ± 1.332 a	1.36 ± 0.060 ab	125.55 ± 41.384 b	107.55 ± 25.479 a	30.62 ± 2.315 ab	67.31 ± 18.666 a

*Data represent average values (n = 5) ± standard error*.

*Different small caps letters indicate significant differences among the different experimental treatments, according to Tukey b or to Dunn-Bonferroni a posteriori tests. Significant effect: p-value ≤ 0.05*.

1 *Data were not analyzed by a two-way ANOVA as data in the non-contaminated soil were below detection limit*.

2 *Data below the detection limit (DL). Not included in the statistical analysis*.

**Table 5:** **(A)** *P*-values of the two-way ANOVA test for the effects of the factors “Copper contamination” and “Mycorrhizal inoculation treatment” and their interaction; **(B)** Macro- and micronutrient contents in leaves of Touriga Nacional grapevine variety plants grafted onto 1103P rootstock inoculated or not with *R. irregulare* or *F. mosseae*.

**(A) Factor**		**Macronutrient**						**Micronutrient**			
		**N**	**P**	**K**	**Na**	**Ca**	**Mg**	**Fe**	**Cu^1^**	**Zn^2^**	**Mn**
Copper contamination		0.003	< 0.0001	0.941	0.003	0.416	0.919	< 0.0001	–	–	< 0.0001
Mycorrhizal inoculation treatment		0.015	< 0.0001	0.472	0.452	0.823	0.916	0.055	–	–	0.031
Interaction		0.398	< 0.0001	0.558	0.072	0.030	0.156	0.126	–	–	0.001
**(B) Soil treatment**	**Inoculation treatment**	**Macronutrient concentraction (g kg**^**−1**^**)**						**Micronutrient concentration (mg kg**^**−1**^**)**			
		**N**	**P**	**K**	**Na**	**Ca**	**Mg**	**Fe**	**Cu**^1^	**Zn**^1^	**Mn**
Non-contaminated	Non-inoculated	13.85 ± 0.902 a	1.25 ± 0.080 b	13.53 ± 0.916 a	0.95 ± 0.080 b	11.93 ± 1.563 a	2.73 ± 0.242 a	111.27 ± 6.502 a	11.08 ± 0.442 a	26.15 ± 0.997 a	54.50 ± 4.119 c
	*R. irregulare*	11.75 ± 0.514 ab	0.81 ± 0.067 c	12.75 ± 0.992 a	1.21 ± 0.088 ab	14.98 ± 1.222 a	3.20 ± 0.197 a	115.83 ± 2.230 a	11.02 ± 0.458 a	< DL ^3^	69.61 ± 9.911 c
	*F. mosseae*	16.62 ± 1.735 a	2.03 ± 0.116 a	11.85 ± 0.884 a	1.16 ± 0.078 ab	11.00 ± 0.818 a	2.56 ± 0.199 a	115.15 ± 6.410 a	11.17 ± 0.585 a	< DL ^3^	67.80 ± 2.740 c
Cu-contaminated soil	Non-inoculated	12.24 ± 0.918 ab	0.54 ± 0.083 d	13.64 ± 1.101 a	1.56 ± 0.178 a	13.37 ± 1.680 a	2.83 ± 0.248 a	78.69 ± 5.779 c	11.11 ± 0.651 a	25.94 ± 0.514 a	290.93 ± 22.480 a
	*R. irregulare*	9.84 ± 0.888 b	0.51 ± 0.035 d	11.11 ± 1.549 a	1.21 ± 0.062 ab	11.44 ± 0.171 a	2.60 ± 0.154 a	83.21 ± 4.007 bc	10.11 ± 0.102 a	25.25 ± 0.220 a	144.03 ± 8.680 b
	*F. mosseae*	11.43 ± 0.359 ab	0.47 ± 0.028 d	13.14 ± 1.581 a	1.58 ± 0.161 a	16.23 ± 2.005 a	3.14 ± 0.436 a	104.25 ± 5.376 ab	14.23 ± 1.729 a	25.86 ± 0.568 a	236.99 ± 16.712 a

*Data represent average values (n = 5) ± standard error*.

*Different small caps letters indicate significant differences among the different experimental treatments, according to Tukey b or to Dunn-Bonferroni a posteriori tests. Significant effect: p-value ≤ 0.05*.

1 *Data were not analyzed by a two-way ANOVA as data did not fulfill a normal distribution and variance homogeneity conditions*.

2 *Data were not analyzed by a two-way ANOVA as in the non-contaminated soil R. irregulare- and F. mosseae-inoculated plants values were below the detection limit*.

3 *Data below the detection limit (DL). Not included in the statistical analysis*.

In the contaminated soil, high levels of Cu led to a general increase in root Cu, as well as in Mg and Mn concentrations, although no differences were detected among inoculation treatments. However, *R. irregulare*-inoculated plants presented an increase in root Zn, K, Fe and Na and a decrease in Ca concentrations (Table 4).

Results indicate that Cu-contaminated soils clearly affect the leaf nutritional status of grapevines, evident as significant differences in the concentrations of key mineral elements. There were significant decreases in N, P and Fe and increases in Na and Mn concentrations in leaves of plants growing in the Cu-contaminated soil (Table 5). The decrease in N and P in leaves was greater in *F. mosseae*-inoculated plants than in non-inoculated or in *R. irregulare*- inoculated plants: with 31.2 vs. 16.3% and 11.7%, decrease respectively in N concentration, and 73 vs. 56.8% and 37.0% in P concentrations (Table 5). Although N and P concentrations were most altered in *F. mosseae*-inoculated plants grown in contaminated soil, these were able to sustain leaf Fe levels relatively unchanged. In contrast, a Fe decrease of nearly 30% was observed in non-inoculated and *R. irregulare*- inoculated plants (Table 5).

The largest alteration in response to soil Cu contamination was observed in leaf Mn concentrations, with the highest increase in the levels of this element in non-inoculated and *F. mosseae*-inoculated plants (5.3-fold and 3.5-fold, respectively). These increases were significantly higher than those observed in *R. irregulare*- inoculated plants, which had a 2.1-fold increase (Table 5). Sodium concentration in leaves was also significantly affected by Cu contamination, particularly in non-inoculated and in *F. mosseae*- inoculated plants, with a 1.6- and a 1.4- fold increase, while *R. irregulare*-inoculated plants were again the ones showing the least alteration (Table 5). No changes were observed in K, Ca, Mg, Cu, and Zn concentrations in response to Cu contamination or to mycorrhizal inoculation treatments.

## Discussion

Plant inoculation with beneficial soil microorganisms such as AMF is increasingly being used in agricultural ecosystems as a way to enhance plant stress tolerance, productivity and sustainability (Gianinazzi et al., 2010; Verbruggen et al., 2013). However, the successful establishment of AMF inoculants in the field depends on several factors, such as soil physico-chemical and biological characteristics, including the resident mycorrhizal community in the soil (Köhl et al., 2016). For this reason, to ensure the establishment of *R. irregulare* and *F. mosseae* inoculants in Touriga Nacional grapevines, one-year old plants obtained from a nursery were pre-inoculated in a sterile substrate. After three months growth in this substrate, root AMF colonization could be observed in all plants, including the non-inoculated ones, indicating that indigenous AMF from the nursery remained and developed in the roots even though these had been previously cut to 2 cm and washed. Unexpectedly, these plants had the highest colonization rates (Figure 1), which may indicate the occurrence of competition within roots of *R. irregulare*- or *F. mosseae*-inoculated plants between the indigenous fungus/fungi and the inoculated AMF, leading to lower colonization rates. Although under natural conditions multiple occupancy could be more beneficial to the host plant (i.e. complementary function of the AMF species) than a single colonization by a single AMF isolate (Jansa et al., 2008; Wagg et al., 2011), competitive interactions have been observed among AMF species (Hepper et al., 1988; Cano and Bago, 2005; Verbruggen et al., 2013; Gijsbert et al., 2015). In fact, mycorrhizal fungi can compete intraradically for host-derived carbon (Thonar et al., 2014), which normally results in reduced root colonization rates (Wilson and Trinick, 1983; Wilson, 1984; Pearson et al., 1994; Cano and Bago, 2005; Engelmoer et al., 2014; Lu et al., 2015). Moreover, additional competitive interactions seem to have taken place when grapevine plants were transplanted to the Arenosol, as mycorrhizal colonization rate was approximately 0.50 in the new roots grown in the non-contaminated Arenosol (Table 2). In this case, competition in the intraradical space would have been between the AMF already colonizing grapevine roots and the resident AMF from the soil, which were present in a concentration of approximately 6.4 mycorrhizal propagules per g. In a parallel study conducted with the same plant genotypes and inoculation treatments but using a previously sterilized soil from the same location, root colonization rates were significantly higher after three months, around 0.80 in all three inoculation treatment plants (non-inoculated, *R. irregulare*- and *F. mosseae*-inoculated plants) (Nogales et al., 2017). Being soil sterilization the unique difference, it seems likely that the reason for the lower colonization rates in the present non-sterile soil is the presence of indigenous AMF.

Regardless of the competition in the intraradical space, plants still benefited from the AMF inoculation, as even if colonization rates were similar in inoculated and non-inoculated plants growing in the non-contaminated Arenosol, root biomass was significantly higher in *R. irregulare*- and *F. mosseae*-inoculated plants (Figure 3C). Furthermore, P concentration in leaves was also highest in *F. mosseae*-inoculated plants, although *R. irregulare*-inoculated plants were in this case the ones showing the lowest content (Table 3). In fact, even though mycorrhizal inoculation commonly increases root and leaf P concentration in grapevines (Schreiner, 2005b; Khalil, 2013), this greatly (although not exclusively) depends on the identity of the fungal partner(s) (Biricolti et al., 1997; Schreiner, 2005a). Apart from P, the rest of the macro- and micronutrients were not affected by the inoculation treatments in the non-contaminated soil. In grapevines, data relying on the role of AMF in the uptake of macro- and micronutrients others than P and N are relatively scarce and sometimes contradictory, which might also be due to the fact that nutritional responses in mycorrhizal grapevines depend greatly on the scion, on the rootstock and on the inoculated AMF (Trouvelot et al., 2015). Therefore, the data from the present study suggest that in this soil, the introduction of *F. mosseae* would be the best option to improve plant P nutrition and root biomass in Touriga Nacional plants grafted onto 1103 Paulsen rootstock.

However, it is important to note that changes in the host's environment, especially abiotic and biotic stresses, can influence greatly the outcome of the symbiosis (Pearson et al., 1994; Hoeksema et al., 2010; Verbruggen et al., 2013), including high soil Cu concentration (Meier et al., 2015). Hence, the evaluation of the effect of high soil Cu concentration in mycorrhizal grapevines, especially in the extractable fraction (considered as available to the organisms), is extremely important for viticulture. In the present study, the concentration of Cu added into the Arenosol attempted to simulate the highest Cu contents observed in Portuguese vineyards (Magalhães et al., 1985; Dias, 1995; Dias and Soveral-Dias, 1997).

Excess copper had various effects on the plants as well as on the soil. Soil dehydrogenase activity, which is an indicator of overall microbial activity and soil quality (Watts et al., 2010; Liang et al., 2014), decreased drastically from 11.9 to 0.67 TTF. g^−1^ three months after Cu addition (Tables 1, 3). However, it needs to be considered that soil dehydrogenase activity measurements can vary greatly depending on the method used and depending on soil properties, especially pH (Januszek et al., 2015). Although Cu can be toxic for soil microorganisms at high concentrations, it is yet unclear whether the soil dehydrogenase activity decrease was due to Cu toxicity, to soil pH decrease (Table 2), which commonly reduces its activity (Januszek et al., 2015), or to a combination of both factors.

On the other hand, root mycorrhizal colonization rate was not affected by high Cu concentration, evident as no differences found in colonization rates between the plants grown in Cu-contaminated and non-contaminated soils. There is some evidence in the literature of decreased mycorrhizal root colonization in plants grown in metal contaminated soils (Karagiannidis and Nikolaou, 2000; Klauberg-Filho et al., 2005; Zhang et al., 2012; Ferrol et al., 2016), while other studies also reveal no changes on this parameter related to soil pollution (Cicatelli et al., 2010).

With Cu addition to the soil, grapevine plants showed a general decrease in NDVI and PRI, as well as in leaf area, shoot length and root biomass, which agrees with other studies in young grapevines grown in Cu-contaminated soils, that also showed reduced root and shoot growth, leaf chlorosis and Cu accumulation in roots (Toselli et al., 2009). However, after three months growth in such soil conditions, differences were observed among plants of the three inoculation treatments, being *F. mosseae*-inoculated plants the ones showing the largest decrease in NDVI and leaf area, and *R. irregulare*-inoculated plants the ones showing the largest decrease in PRI and root biomass (Figures 2, 3). Non-inoculated plants were the less affected by Cu contamination, especially in terms of root growth (Figure 3C).

As observed for vegetative parameters, the effect of AMF inoculation on plant nutrition under high soil Cu concentrations was different from the one observed under non-contaminated soil conditions. This agrees with Meier et al. (2015), who found that the effect of different mycorrhizal treatments on the levels of shoot nutrients depended on soil Cu concentration. Ambrosini et al. (2015) observed that although mycorrhizal inoculation of grapevines in soils with high soil Cu concentrations induced root accumulation of this element, translocation to leaves depended on the inoculated species, and was only observed in plants inoculated with *Rhizoglomus clarus* and *Acaulospora morrowiae*. In Touriga Nacional plants grafted onto 1103 Paulsen rootstock, Cu contamination led to an accumulation of this element in roots, but no translocation to leaves was observed. Besides, roots and shoots from all three mycorrhizal inoculation treatments presented similar Cu contents (Tables 4, 5).

At high concentrations, Cu commonly interacts with other elements. These interactions can be antagonistic or synergistic, as Cu can inhibit or stimulate the absorption of other elements in plants, leading to imbalanced reactions that may cause a chemical stress in plants and a consequent growth decrease (Kabata-Pendias, 2010). In the present study, Cu addition in the soil induced an increase in the bioavailability of Mn and Fe in the soil, and a general increase in root Cu, Mn and Mg contents (Tables 2, 4). Thus, the altered concentration of these elements in roots might have contributed to the root growth decrease observed in plants grown in the contaminated soil. Moreover, plants inoculated with *R. irregulare* showed as well altered levels of other nutrients in roots such as an increase in Zn, Fe, K and Na, and a decrease in Ca concentration (Table 4), which may have contributed to the higher decrease in root biomass observed in these plants.

Even though Cu contamination induced a general increase in the concentration of these elements in roots, only Mn was translocated from the soil to the leaves, while Cu itself remained accumulated in roots. Although Mn uptake is metabolically controlled, when present at high available soil concentrations, its uptake may also be passive and can be rapidly translocated within plants (Kabata-Pendias, 2010). This could explain the large Mn increase found in roots and leaves in grapevines, which was particularly high in non-inoculated and *F. mosseae*-inoculated plant leaves (Table 5). Manganese shows antagonistic interactions with Fe, and therefore, when Mn is in excess, it competes with Fe for binding sites in enzymes and can interfere with its transport from roots to shoots (Srivastava and Gupta, 1996), leading to Fe deficiency. In general, an appropriate level of both elements is needed for a healthy plant growth (Fe:Mn ratio should be between 1.5 and 2.5), as both elements are interrelated in their metabolic functions (Kabata-Pendias, 2010). Thus, an increase of Mn leads to low Fe:Mn ratios and subsequent symptoms of Fe deficiency and Mn toxicity. Those are characterized by chlorosis and brown spots on leaves, where Mn is concentrated (Kabata-Pendias, 2010). These symptoms were similar to the ones observed in our plants, especially in the non-inoculated ones (Figure 4). In fact, the lowest Fe:Mn ratio was observed in these ones (0.27), followed by *F. mosseae*- inoculated (0.44) and *R. irregulare*-inoculated plants (0.58).

Excess Cu in the soil can also interfere directly in the absorption and translocation of Fe into the plant, resulting in a decrease of chlorophyll content (Kabata-Pendias, 2010) that appears as yellowing or chlorosis in the interveinal areas of the emerging leaves and that later expands until the entire leaf turns yellow and finally white (Treeby et al., 2004). It seems therefore that the interveinal chlorosis observed in young leaves of Touriga Nacional plants grown in the contaminated soil (Figure 4) and the decrease in leaf Fe concentration (Table 5) can be due to a negative interaction of leaf Fe content with high concentrations of Cu in roots and with Mn in roots and leaves. The largest decreases in Fe content in leaves were observed in non-inoculated and *R. irregulare*-inoculated plants (29 and 28%, respectively, vs. 9% in *F. mosseae*-inoculated plants). In fact, according to Romero et al. (2014), Fe levels would be below the optimal levels recommended for grapevines in non-inoculated and an in *R. irregulare*-inoculated plants (below 99 mg kg^−1^).

On the other hand, the decrease in leaf P and N concentration does not seem to be related to a decrease in both nutrient uptakes, as both element concentrations in roots were not affected by Cu contamination. This is in contrast to the study of Melo et al. (2008) who observed that as soil Cu concentration increased in an Udorthent soil (with low organic matter content) N and P concentrations decreased in 1103 Paulsen plant roots (*R*^2^ = 0.52 and 0.38, respectively). However, in leaves, they also found a linear decrease in N and P contents as Cu concentration increased in the soil (Melo et al., 2008), while Toselli et al. (2009) observed a decrease in leaf P concentration but not a decrease in N in young potted grapevine *cv*. Sangiovese plants grafted onto SO4. In Touriga Nacional plants, the observed decrease in leaf P concentration could be related to an antagonistic interaction between Mn and P, as reductions in plant P with increasing Mn, especially at very high Mn concentrations, have also been observed in other plant species (Gunes et al., 1998; Sarkar et al., 2004).

Copper and Zn interactions are also commonly observed because both metals seem to be absorbed by the same mechanism and therefore each may competitively inhibit root absorption of the other (Kabata-Pendias, 2010). However, in the present study, no antagonism was observed between both elements, as at high soil Cu concentrations the concentrations of both elements in roots increased and remained unchanged in leaves (Tables 4, 5). This is in agreement to Toselli et al. (2009), who also found that high soil Cu concentrations did not alter leaf Cu and Zn contents in young potted vines *cv*. Sangiovese, although they reported an antagonistic interaction among both elements in roots.

Several studies suggest that the ability of AMF species to mitigate the stress caused by soil Cu contamination in plants is due to a promotion in the absorption of water and nutrients (Soares and Siqueira, 2008; Andrade et al., 2010), specially P (Ambrosini et al., 2015). Nonetheless, in the present study, no clear relation was observed between a better plant nutrition and a better tolerance of Touriga Nacional plants to high Cu soil concentrations in terms of growth. Indeed, *R. irregulare*-inoculated plants were the ones sustaining better the decrease in P and the increases in Mn and Na contents in leaves, but they were, together with *F. mosseae*-inoculated plants, the ones showing the largest decrease in total leaf areas and root biomass. By contrast, non-inoculated plants were the ones sustaining better their growth in the contaminated soil, but they were the ones showing the highest increase in Mn and Na in leaves.

The better growth response of non-inoculated plants to the high Cu level in the available soil fraction when compared to the AMF inoculated plants could be related to the fact that both *R. irregulare* and *F. mosseae* isolates used in this study were highly infective and fast growing (both isolates are sold commercially). Consequently, their carbon demand to the plant might be higher than the one of the indigenous species from the nursery and from the Arenosol, and in such stressful environment where plant's carbon availability is commonly reduced, this might result in a growth decrease. In fact, AMF may express different symbiotic lifestyles depending on environmental factors and stress conditions, on host genotypes or on their physiological status. Based on these factors, AMF may establish mutualistic symbioses, commensalistic symbioses (providing no benefit to the plant), or parasitic symbioses (resulting in a negative growth response, as observed when the costs of the symbiosis exceed the nutritional benefits to the host) (Johnson and Graham, 2013). It is clear therefore, that soil chemical composition is an important factor to take into account when selecting the most appropriate AMF to inoculate grapevines.

## Conclusions

Although grapevine plants benefited from the inoculation with *R. irregulare* and *F. mosseae* during development in an Arenosol with normal Cu concentration, inoculation with those AMF revealed to be non-efficient or even detrimental to grapevine growth in the same soil with high Cu concentration in the available fraction. In this soil, the native AMF present in the non-inoculated plants, proceeding from the plant nursery and/or the field, were more efficient in sustaining shoot and root growth. However, considering plant nutritional status, *R. irregulare*-inoculated plants were the ones better avoiding Mn increase in leaves, and *F. mosseae*-inoculated plants were the ones that better sustained leaf Fe levels. As grapevine nutritional status is crucial for obtaining a good wine quality, these parameters need to be considered when deciding about the suitability of field AMF inoculation for a particular grapevine genotype.

It remains clear therefore that soil environment, especially the occurrence of soil contaminants such as Cu, and the presence of native AMF are crucial factors to consider before grapevine mycorrhizal inoculation in vineyards. Also importantly, inoculation procedures should deserve special attention due to potential competition between AMF species/isolates in the soil, as here demonstrated.

## Author Contributions

AN, MMA, HSP, CL, and WV conceived and designed research. AN and ES conducted experiments. AN, ES, DA, and GV analyzed and interpreted the results. AN wrote the manuscript and all authors read and approved the manuscript.

### Conflict of Interest Statement

The authors declare that the research was conducted in the absence of any commercial or financial relationships that could be construed as a potential conflict of interest.
